# MED12 and CDK8/19 Modulate Androgen Receptor Activity and Enzalutamide Response in Prostate Cancer

**DOI:** 10.1210/endocr/bqae114

**Published:** 2024-09-10

**Authors:** Chiara Andolfi, Caterina Bartolini, Elisa Morales, Büşra Gündoğdu, Martin Puhr, Juan Guzman, Sven Wach, Helge Taubert, Achim Aigner, Iris E Eder, Florian Handle, Zoran Culig

**Affiliations:** Department of Urology, Division of Experimental Urology, Medical University of Innsbruck, 6020 Innsbruck, Austria; Department of Urology, Division of Experimental Urology, Medical University of Innsbruck, 6020 Innsbruck, Austria; University of Florence, 50 121 Florence, Italy; Department of Urology, Division of Experimental Urology, Medical University of Innsbruck, 6020 Innsbruck, Austria; Johannes Gutenberg University Mainz, 55122 Mainz, Germany; Department of Urology, Division of Experimental Urology, Medical University of Innsbruck, 6020 Innsbruck, Austria; Graudate School of Science and Engineering, Yıldız Technical University, 34220 Istanbul, Turkey; Department of Urology, Division of Experimental Urology, Medical University of Innsbruck, 6020 Innsbruck, Austria; Department of Urology and Pediatric Urology, Universitätsklinikum Erlangen, 91054 Erlangen, Germany; Department of Urology and Pediatric Urology, Universitätsklinikum Erlangen, 91054 Erlangen, Germany; Department of Urology and Pediatric Urology, Universitätsklinikum Erlangen, 91054 Erlangen, Germany; Rudolf-Boehm-Institute for Pharmacology and Toxicology, Clinical Pharmacology, University of Leipzig, 04107 Leipzig, Germany; Department of Urology, Division of Experimental Urology, Medical University of Innsbruck, 6020 Innsbruck, Austria; Department of Urology, Division of Experimental Urology, Medical University of Innsbruck, 6020 Innsbruck, Austria; Institute of Pathology, Neuropathology & Molecular Pathology, Medical University of Innsbruck, 6020 Innsbruck, Austria; Department of Urology, Division of Experimental Urology, Medical University of Innsbruck, 6020 Innsbruck, Austria

**Keywords:** prostate cancer, Mediator complex, androgen receptor, enzalutamide

## Abstract

Prostate cancer progression is driven by androgen receptor (AR) activity, which is a target for therapeutic approaches. Enzalutamide is an AR inhibitor that prolongs the survival of patients with advanced prostate cancer. However, resistance mechanisms arise and impair its efficacy. One of these mechanisms is the expression of AR-V7, a constitutively active AR splice variant. The Mediator complex is a multisubunit protein that modulates gene expression on a genome-wide scale. MED12 and cyclin-dependent kinase (CDK)8, or its paralog CDK19, are components of the kinase module that regulates the proliferation of prostate cancer cells. In this study, we investigated how MED12 and CDK8/19 influence cancer-driven processes in prostate cancer cell lines, focusing on AR activity and the enzalutamide response. We inhibited MED12 expression and CDK8/19 activity in LNCaP (AR^+^, enzalutamide-sensitive), 22Rv1 (AR-V7^+^, enzalutamide-resistant), and PC3 (AR^−^, enzalutamide-insensitive) cells. Both MED12 and CDK8/19 inhibition reduced cell proliferation in all cell lines, and MED12 inhibition reduced proliferation in the respective 3D spheroids. MED12 knockdown significantly inhibited c-Myc protein expression and signaling pathways. In 22Rv1 cells, it consistently inhibited the AR response, prostate-specific antigen (PSA) secretion, AR target genes, and AR-V7 expression. Combined with enzalutamide, MED12 inhibition additively decreased the AR activity in both LNCaP and 22Rv1 cells. CDK8/19 inhibition significantly decreased PSA secretion in LNCaP and 22Rv1 cells and, when combined with enzalutamide, additively reduced proliferation in 22Rv1 cells. Our study revealed that MED12 and CDK8/19 regulate AR activity and that their inhibition may modulate response to enzalutamide in prostate cancer.

Prostate cancer is driven by the androgen receptor (AR), an androgen-dependent transcription factor that promotes cell growth and proliferation (1). Castration-resistant prostate cancer (CRPC) is an advanced form of the disease in which cancer cells can survive despite androgen deprivation, frequently by restoring AR activity (2). Second-generation AR signaling inhibitors, such as enzalutamide, suppress AR activity by competitively inhibiting androgen binding, thereby significantly prolonging CRPC patient survival (3, 4). However, cancer cells ultimately develop resistance to enzalutamide (5). One major mechanism of enzalutamide resistance is the enhanced expression of AR-V7, a constitutively active splice variant of AR that restores AR activity in patients treated with AR signaling inhibitors (6, 7). Other mechanisms, such as the overexpression of AR coactivators, also contribute to the impairment of enzalutamide efficacy (5).

Some subunits of the Mediator complex are coactivators of the AR in prostate cancer (8, 9). The Mediator complex is a multisubunit protein that modulates gene expression on a genome-wide scale. It comprises the preinitiation complex on gene core promoters, where it mediates the interaction between RNA polymerase II and enhancer-bound transcription factors, thus promoting transcription initiation (10, 11). The Mediator complex has a modular structure with the head, middle, and tail modules acting as the main core to which a kinase module can transiently associate (10). The kinase module is a key Mediator complex regulator that can disrupt its interaction with RNA polymerase II and phosphorylate transcription factors, consequently affecting their activity and stability (12-14).

MED12 is an essential subunit of the kinase module that plays a crucial role in preserving the structure of the entire module (15-17). Adler et al showed that MED12 is overexpressed in advanced CRPC compared to early-stage disease (18). They also related MED12 knockdown to decreased cell proliferation and reduced transition to the S stage of the cell cycle (18). Recent findings have shown that MED12 downregulation increased the proliferation of prostate cancer cells under androgen deprivation (19).

Cyclin-dependent kinase (CDK)8 and its alternative paralog, CDK19, are the enzymatic subunits of the kinase module of the Mediator complex (15). In prostate cancer cells, CDK8/19 inhibition downregulates cell proliferation and in vivo metastasis formation (20, 21). Offermann et al demonstrated that combining CDK8/19 with bicalutamide, an AR inhibitor, additively decreased the proliferation of prostate cancer cells (22).

Taken together, these results suggest that MED12 and CDK8/19 play important roles in promoting tumorigenesis and resistance to therapy in prostate cancer cells. Our study investigated how the inhibition of MED12 and CDK8/19 subunits affects cancer signaling pathways and cell processes in prostate cancer, focusing on AR activity and cell responsiveness to enzalutamide.

## Methods

### Cell Lines

LNCaP (RRID: CVCL_1379), 22Rv1 (RRID: CVCL_1045), and PC3 (RRID: CVCL_0035) cells were acquired from the American Type Culture Collection. All cells were cultured in RPMI-1640 medium supplemented with 10% fetal bovine serum (PAN-Biotech, #P30-3031), 1% penicillin/streptomycin (PAN-Biotech, #P06-07100), and 1% GlutaMAX (Thermo Fisher Scientific, #51985026). Cells were cultured at 37 °C and 5% CO_2_ in a humidified atmosphere. All the cell lines were subjected to regular mycoplasma testing. Multiwell plates were coated with poly-D-lysine (Sigma-Aldrich, #P6407) before seeding LNCaP cells. Cell line authentication was performed using short tandem repeat analysis.

### Small Interfering RNA-Mediated Transfection

Cells were seeded in 6-well plates (LNCaP and 22Rv1: 300 000 cells/well; PC3: 250 000 cells/well) and incubated for 24 hours. Cells were transfected with 25 pmol ON-TARGETplus SMARTpools (Dharmacon, Horizon Discovery) [siCtrl: nontargeting pool, #D-001810-10-05; siMED12: human MED12 small interfering RNA (siRNA)], #L-009092-00-0005) and 7.5 µL of Lipofectamine RNAiMAX (Thermo Fisher Scientific, #13778150). Transfection was performed according to the manufacturer's instructions. The experimental endpoints were set at 3 and 6 days after transfection for 2D cell lines and at 7 days for 3D models (spheroids).

### Cell Proliferation Assay

The proliferation of adherent cells seeded in 6-well plates was evaluated 3 and 6 days after transfection. At both end time points, the cells were incubated with Hoechst 333423342 nucleic acid stain (Thermo Fisher Scientific, #H3570) at a 1:2000 dilution for 20 minutes. Cell nuclei were visualized using fluorescence imaging (CELENA^®^ S Digital Imaging System, Logos Biosystems) and counted using the Fiji ImageJ software.

### Formation and Analysis of Spheroids

Three days after siRNA transfection, spheroids were generated in 96-well plates (Corning^®^ 96 Well Clear Round Bottom Ultra Low Attachment Microplate, Corning, #COR7007), and all downstream analyses were performed after 4 days. Spheroids were harvested and lysed with 25 µL 1× DNA/RNA Shield (Zymo Research, #R1100-50). Cell number estimation was performed by measuring the total DNA content of spheroids using the Qubit 1× dsDNA Assay Kit (Thermo Fisher Scientific, #Q33230) and comparing it with a standard known cell number.

### RNA Extraction and Reverse Transcription-Quantitative PCR

Total RNA was extracted from the cells using the EXTRACTME TOTAL RNA KIT (#EM09.2, Blirt S.A.) and quantified using a NanoDrop^™^ 2000/2000c (Thermo Fisher Scientific, #ND2000CLAPTOP). Total RNA from the spheroids was extracted using the Quick-RNA MagBead kit (Zymo, #R2132).

cDNA was synthesized using the LunaScript^®^ RT SuperMix Kit (New England Biolabs, #M3010X), and gene expression was quantified by quantitative PCR (qPCR) using the Luna^®^ Universal qPCR Master Mix (New England Biolabs, #M3004X) and the CFX Connect Real-Time PCR Detection System (Bio-Rad, #1855200). qPCR was performed according to the manufacturer's protocol using the TaqMan gene expression assays listed in Supplementary Table 1 (23).

### Prostate-Specific Antigen Quantification

Prostate-specific antigen (PSA) secreted by cells in the medium was quantified using chemiluminescent-based Elecsys total PSA assay (Roche, #04641655 190) and measured on a Cobas^®^ 8000 machine (Roche).

### Western Blot

Protein isolation from the cell pellets was performed in radioimmunoprecipitation assay lysis buffer containing a 5% protease inhibitor cocktail (Merck KGaA, #539134). Protein lysates were quantified using the Bradford protein assay and electrophoretically separated on Bolt^TM^ Bis-Tris Plus Mini Protein Gels (Thermo Fisher Scientific, #NW04122BOX). The proteins were blotted on Amersham™ Protran^®^ nitrocellulose membranes with 0.25 µM pore size (Merck, #10600001). Membranes were blocked within 5% skimmed milk powder (#T145.2, Carl Roth) and incubated with primary antibodies (Table 1). IRDye goat anti-rabbit (RRID: AB_10956166, #926-68071, 1:5000) and anti-mouse (RRID: AB_621842, #926-32210, 1:5000) immunoglobin G secondary antibodies (LI-COR Biosciences) were used to detect the AR, AR-V7, and glyceraldehyde-3-phosphate dehydrogenase signals. Enhanced chemiluminescent horseradish peroxidase-conjugated secondary antibodies (RRID: AB_2313567, Jackson ImmunoResearch, #111-035-003, 1:100 000) were used to detect MED12 and c-MYC signals. Signal detection was performed using the Odyssey CLx near-infrared imager (LI-COR Biosciences) for fluorescent antibodies or using SuperSignal West Femto Maximum Sensitivity ECL Substrate (Thermo Fisher Scientific, #11859290) and ChemiDoc MP (Bio-Rad) for horseradish peroxidase-conjugated antibodies. Signal quantification was performed using the ImageStudio software (version 5.2, LI-COR Biosciences).

**Table 1.** List of primary antibodies

Protein target	Manufacturer	Catalog number	RRID	Dilution	Clonality, species
MED12	Cell Signaling Technology	143604360	AB_2798461	1:1000	Monoclonal, rabbit
c-MYC	Cell Signaling Technology	5605	AB_1903938	1:1000	Monoclonal, rabbit
AR	Cell Signaling Technology	5153	AB_10691711	1:1000	Monoclonal, rabbit
AR-V7	RevMAb Bioscience	31-1109-00	AB_2716436	1:1000	Monoclonal, rabbit
CDK8	Cell Signaling technology	173957395S	AB_2798784	1:1000	Monoclonal, rabbit
CDK19	Santa Cruz Biotechnology	sc-517026	n.a.	1:1000	Monoclonal, mouse
GAPDH	Millipore	MAB374	AB_2107445	1:50 000	Monoclonal, mouse

Abbreviations: AR, androgen receptor; CDK, cyclin-dependent kinase; GAPDH, glyceraldehyde-3-phosphate dehydrogenase RRID, Research Resource Identifier.

### Enzalutamide Treatment

LNCaP and 22Rv1 cells were treated with 10 µM enzalutamide (MedChemExpress, #HY-70002) and the respective dimethylsulfoxide volumes immediately after siRNA transfection and medium change. After 3 days, the cells were trypsinized and reseeded. Downstream analyses were performed 6 days after enzalutamide treatment and siRNA transfection.

### Dose–Response Assay

Cells were seeded in 96-well plates at a density of 8000 cells/well. After 24 hours, the cells were treated with sequential 1:2 dilutions of a CDK8/19 inhibitor, SEL120-34A (Selleck Chemicals #S8840), at a concentration range of 0.47 to 15 µM. After 3 days, the cells were lysed in 1× Tris-acetate-EDTA buffer containing 1× TritonX-100 and 1% Proteinase K (Roth Industries, #7528.2). The total amount of DNA was measured by incubating the cell lysates with 1:1000 SYBR^®^ Green I nucleic acid gel stain (Sigma Aldrich, #S9430) for 10 minutes. The fluorescent signal was detected using a Cytation 5 Imaging Reader (BioTek Instruments). Cell number was calculated from the amount of DNA using a known standard. The IC50 values for cell proliferation for each cell line were calculated using GraphPad Prism 10.1.2 (RRID: SCR_002798).

### Population Doubling Level (Programmed Death Ligand) Assay

22Rv1 cells were seeded into T25 cell culture flasks and treated with single or combined treatments of 10 µM enzalutamide (MedChemExpress, #HY-70002) and 1.5 µM SEL120-34A (Selleck Chemicals, #S8840) for 18 days. The cells were passaged regularly into new flasks. At each passage, the cells were counted using the Schärfe Casy TT system. The cumulative programmed death ligand (PDL) was calculated using the formula: $3.32\times (\mathrm{lo}g_{10}(harvested\;cells)-\mathrm{lo}g_{10}(seeded\;cells))+previous\;cumulative\;PDL.$ The experiments were performed in a single replicate.

### Gene Correlation Analysis

Gene correlations were analyzed in publicly available prostate cancer tissue databases (TCGA_PRAD, GSE62872, GSE21034, GSE46691, and SU2C_PRAD) as previously described (24). The first 4 databases contain primary prostate cancer tissues, whereas the SU2C_PRAD database contains CRPC tissues, most of which are metastatic.

### Gene Number Quantification

MED12 copy number aberration was extracted from the TCGA_PRAD and SU2C datasets.

### Cell Line Dependency to MED12 Expression

The dependence of a benign prostatic hyperplasia cell line (BPH1) and prostate cancer cell lines (LNCaP, 22Rv1, VCaP, DuCaP, DU145, and PC3) on MED12 expression was analyzed using 2 independent publicly available screens from the DepMaP portal (RRID: SCR_017655). MED12 expression was downregulated using CRISPR or RNA interference (RNAi) and its effect on cell growth was measured. In the plot, complete cell dependence is located at ‘−1’ on the *x*-axis, whereas complete cell independence is located at 0.

### RNA Sequencing and Pathway Analysis

High-throughput 3′ RNA sequencing (RNA-seq) was performed using a BRB-seq Library Preparation Kit for Illumina (Alithea Genomics). Read alignment and counting were performed with STARsolo (SCR_021542) using Gencode Release 43 (GRCh38.p13) genome assembly. Strand-specific full transcriptome RNA-seq of the 22Rv1 samples was performed using NovoGene. Read alignment and counting were performed in the R software using the Rsubread package and the Gencode Release 43 (GRCh38.p13) genome assembly. Differential gene expression analysis was performed using the edgeR package in the R software. Pathway analysis (camera function) was performed using MSigDB hallmark gene sets version 7.5.1.

### Statistics

Unpaired *t*-tests were used to analyze the differences between treated samples and their relative controls. A simple linear regression was applied to the PDL assay. Statistical significance was always considered as a *P*-value < .05 (*P < .05, **P < .01, ***P < .001). Data are presented as the mean ± SD. All experiments were performed in at least 3 biological replicates, unless otherwise noted.

## Results

### MED12 Knockdown Inhibits Prostate Cancer Cell Proliferation

**Figure 1.**
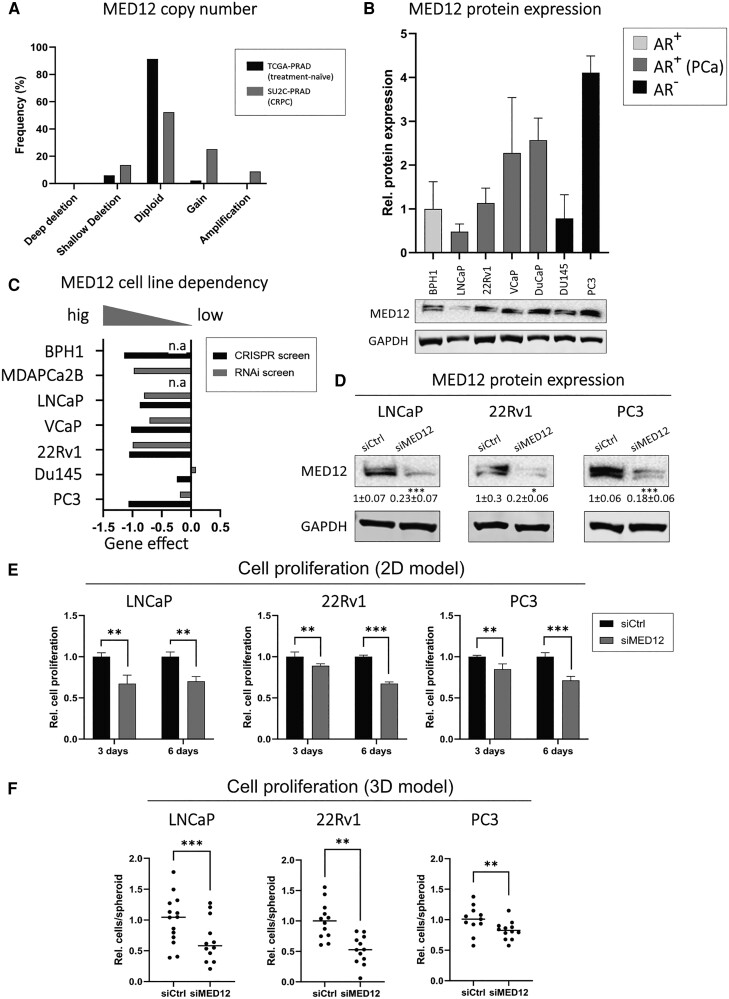
MED12 knockdown inhibits prostate cancer cell proliferation. (A) MED12 gene copy number in cohorts of primary prostate cancer (TCGA-PRAD) and castration-resistant prostate cancer (SU2C-PRAD) tissues. (B) MED12 protein quantification in a benign prostatic hyperplasia cell line (BPH1) and prostate cancer cell lines (LNCaP, 22Rv1, VCaP, DuCaP, Du145, and PC3). A representative Western blot is shown for each cell line. (C) RNA interference and CRISPR screens evaluating prostate cancer cell dependency on MED12 expression for their growth. Lower scores correspond to a higher dependency: a score of 0 is equivalent to nonessential genes and a score of −1 is equivalent to the median of essential genes. (D) MED12 protein quantification in LNCaP, 22Rv1, and PC3 cells transfected with siCtrl and siMED12. A representative Western blot is shown for each cell line. (E) Fluorescent-based cell number quantification of LNCaP, 22Rv1, and PC3 cells transfected with siCtrl and siMED12. (F) Fluorescent-based quantification of the total cell number in LNCaP, 22Rv1, and PC3 spheroids formed after pretransfection of siMED12 and siCtrl in 2D cells (total transfection time: 7 days). Data are represented as mean ± SD. *P < .05, **P < .01, ***P < .001.

To investigate the role of MED12 in prostate cancer, we analyzed aberrations in the MED12 gene copy number status in 2 publicly available datasets: treatment-naïve (TCGA-PRAD, primary prostate cancer) and CRPC (SU2C-PRAD, predominantly metastatic prostate cancer) tissues. The MED12 copy number was increased in over 30% of CRPC samples, compared to less than 3% of treatment-naïve prostate cancer samples (Fig. 1A). Subsequently, we analyzed MED12 protein expression in a panel of prostate cancer cell lines (AR-positive: LNCaP, 22Rv1, VCaP, and DuCaP; AR-negative: DU145 and PC3) and in 1 benign prostatic hyperplasia cell line (BPH1) (Fig. 1B). MED12 protein expression was highly variable among the different prostate cancer cell lines. Consistent with the copy number data, we observed that 3 out of the 6 prostate cancer cell lines showed a strong increase in MED12 protein expression (>2× compared to benign BPH1 cells). LNCaP cells had the lowest MED12 protein levels. In addition, 2 independent datasets from CRISPR- and RNAi-based screens (DepMap portal) revealed that nearly all prostate cancer cell lines were highly sensitive to MED12 downregulation, suggesting its pivotal role in prostate cancer cell growth and survival. Of note, AR-positive cell lines (VCaP, LNCaP, MDA PCa 2b, and 22RV1) were more sensitive to MED12 downregulation than AR-negative cells (PC3 and DU145) in the RNAi-based screen (Fig. 1C).

Based on these findings, we investigated the role of MED12 in prostate cancer cell models resembling various cancer stages and MED12 expression levels. We selected LNCaP (low MED12 expression, androgen-dependent, and enzalutamide-sensitive), 22Rv1 (intermediate MED12 expression, androgen-responsive but enzalutamide-resistant), and PC3 (high MED12 expression, AR-negative, and enzalutamide-insensitive) cell lines. RNAi-mediated MED12 knockdown was highly efficient at both the mRNA and protein levels [Fig. 1D, Supplementary Fig. S1 (25)] posttransfection endpoints. MED12 knockdown significantly decreased cell proliferation, with the most pronounced effect observed after 6 days (LNCaP: −30%, *P* = .003; 22Rv1: −33%, *P* < .001; PC3: −29%, *P* < .001) (Fig. 1E). Similar effects were observed in 3D prostate cancer spheroids generated after pretreatment with RNAi in 2D cell cultures. MED12 knockdown was highly efficient in the spheroids [Supplementary Fig. S1 (25)], in which it significantly decreased cell proliferation (LNCaP: −34%, *P* < .001; 22RV1: −48%, *P* = .002; PC3: −18%; *P* = .004) (Fig. 1F). Taken together, all tested prostate cancer cell lines were susceptible to MED12 knockdown in both 2D and 3D settings.

### MED12 Knockdown Downregulates c-Myc Signaling and Protein Expression

**Figure 2.**
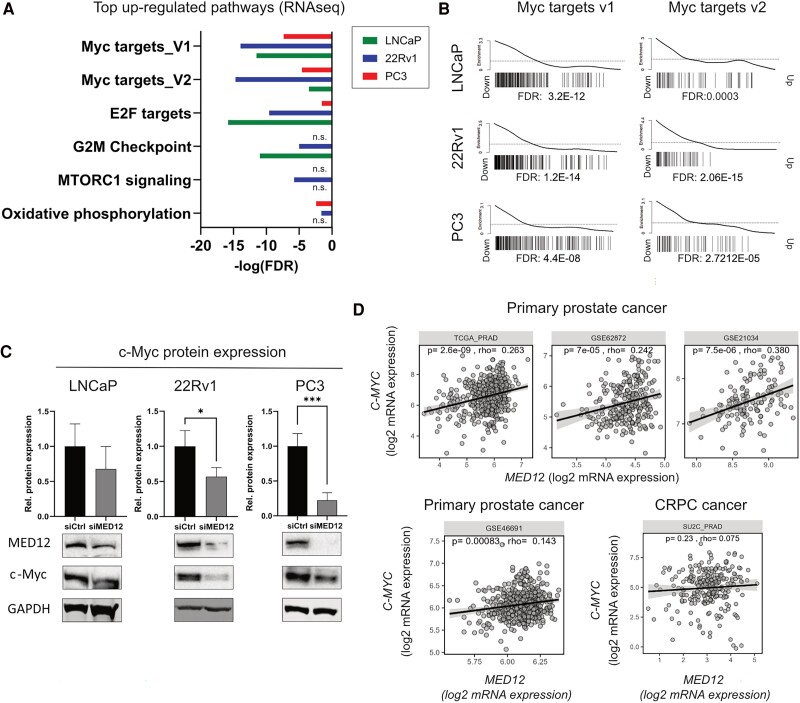
MED12 knockdown downregulates c-Myc signaling and protein expression. (A) Top MSigDB hallmarks related to cell proliferation and significantly altered in LNCaP, 22Rv1, and PC3 cells after MED12 knockdown (false discovery rate < 0.05; transfection time: 3 days). (B) Barcode plot of Myc targets V1 and V2 in LNCaP, 22Rv1, and PC3 cells after MED12 knockdown. (C) c-Myc protein expression in control and siMED12-transfected LNCaP, 22Rv1, and PC3 cells (transfection time: 3 days). A representative Western blot is shown for each cell line. Data are presented as mean ± SD. Statistical significance: *P < .05, **P < .01, ***P < .001. (D) MED12 and c-MYC gene expression correlation in cohorts of primary prostate cancer (TCGA_PRAD, GSE62872, GSE21034, GSE46691) and castration-resistant prostate cancer tissues (SUC2_PRAD).

To further explore the role of MED12 in prostate cancer, we performed a high-throughput RNA-seq gene expression study to evaluate the effect of MED12 knockdown on the cancer-promoting pathways in LNCaP, 22Rv1, and PC3 cells [Fig. 2A, Supplementary Fig. S2 (25)]. The MYC targets v1 and v2 gene sets (26) were significantly downregulated in all 3 cell lines (Fig. 2A and 2B), as well as in 22Rv1 spheroids [Supplementary Fig. S2 (25)]. c-Myc is a nuclear transcription factor that strongly promotes multiple malignant processes in prostate cancer, including cell proliferation and invasion (26). MED12 downregulation significantly reduced c-Myc mRNA [Supplementary Fig. S3 (25)] and protein expression in 22Rv1 and PC3 cells (Fig. 2C, 22Rv1: −43%, *P* = .04; PC3: −83%, *P* < .001). A similar trend was observed in LNCaP cells, although it was not statistically significant [Fig. 2C, Supplementary Fig. S3 (25)]. These findings align with those of 4 cohorts of primary prostate cancer tissues (TCGA_PRAD, GSE62872, GSE21034, and GSE46691), which showed a significant positive correlation between *MED12* and *c-MYC* mRNA expression (Fig. 2D). This correlation was not observed in the cohort of CRPC tissues (SU2C_PRAD) (Fig. 2D).

### MED12 Knockdown Decreases AR Activity

**Figure 3.**
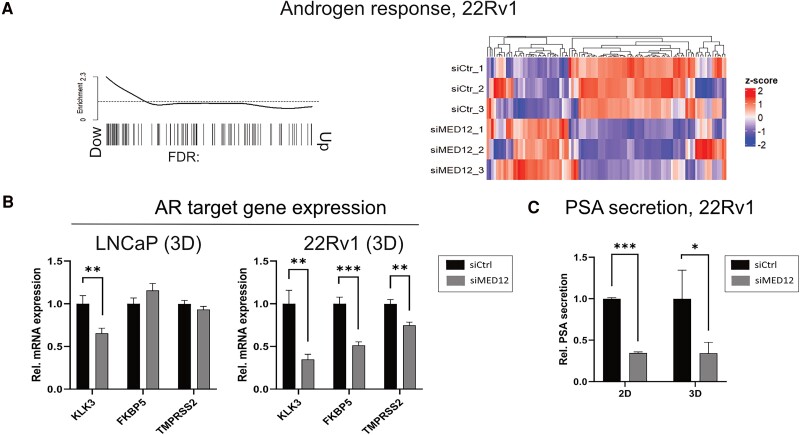
MED12 knockdown decreases androgen receptor activity. (A) Barcode plot and batch-corrected heatmap of androgen response in 22Rv1 cells after MED12 knockdown (transfection time: 3 days). Statistical significance: false discovery rate < 0.05. (B) Quantitative reverse transcription polymerase chain reaction quantification of *KLK3, FKBP5,* and *TMPRSS2* mRNA expression in LNCaP and 22Rv1 3D models (transfection time: 7 days). All data are presented as mean ± SD. (C) Quantification of prostate-specific antigen protein secreted by 22Rv1 in cell medium following MED12 knockdown (chemiluminescent-based assay). Statistical significance: *P < .05, **P < .01, ***P < .001.

In the high-throughput RNA-seq data, we observed that MED12 knockdown strongly deregulates individual genes within the hallmark androgen response gene set in AR-positive cell lines, although the gene set as a whole did not reach statistical significance [Supplementary Fig. S4 (25)]. Therefore, we performed a more in-depth full transcriptome RNA-seq analysis, which revealed that MED12 loss significantly downregulated the androgen response gene set in 22Rv1 cells (Fig. 3A). However, MED12 knockdown had an opposite effect on some individual genes. We quantified the mRNA expression of the AR target genes *KLK3, FKBP5,* and *TMPRSS2* in LNCaP and 22Rv1 3D spheroids using reverse transcription-qPCR (Fig. 3B). *KLK3* mRNA expression was significantly downregulated in LNCaP 3D spheroids (−35%, *P* = .006), whereas the expression of other genes was unaffected. MED12 knockdown inhibited the androgen response in 22Rv1 cells, where it significantly downregulated the mRNA expression of all the tested AR target genes (*KLK3*: −65%, *P* = .002; *FKBP5*: −49%, *P* = .0006; *TMPRSS2*: −26%, *P* = .001) (Fig. 3B). For confirmatory analysis, we quantified the secretion of KLK3 protein product (PSA), which was significantly downregulated in 22Rv1 2D (−66%, *P* < .001) and 3D models (−66%, *P* = .04) (Fig. 3C).

Altogether, our results suggest that MED12 downregulates AR activity in 22Rv1 cells, whereas its role in LNCaP cells is less prominent.

### MED12 Knockdown Inhibits AR-V7 Protein Expression and Modifies Response to Enzalutamide

**Figure 4.**
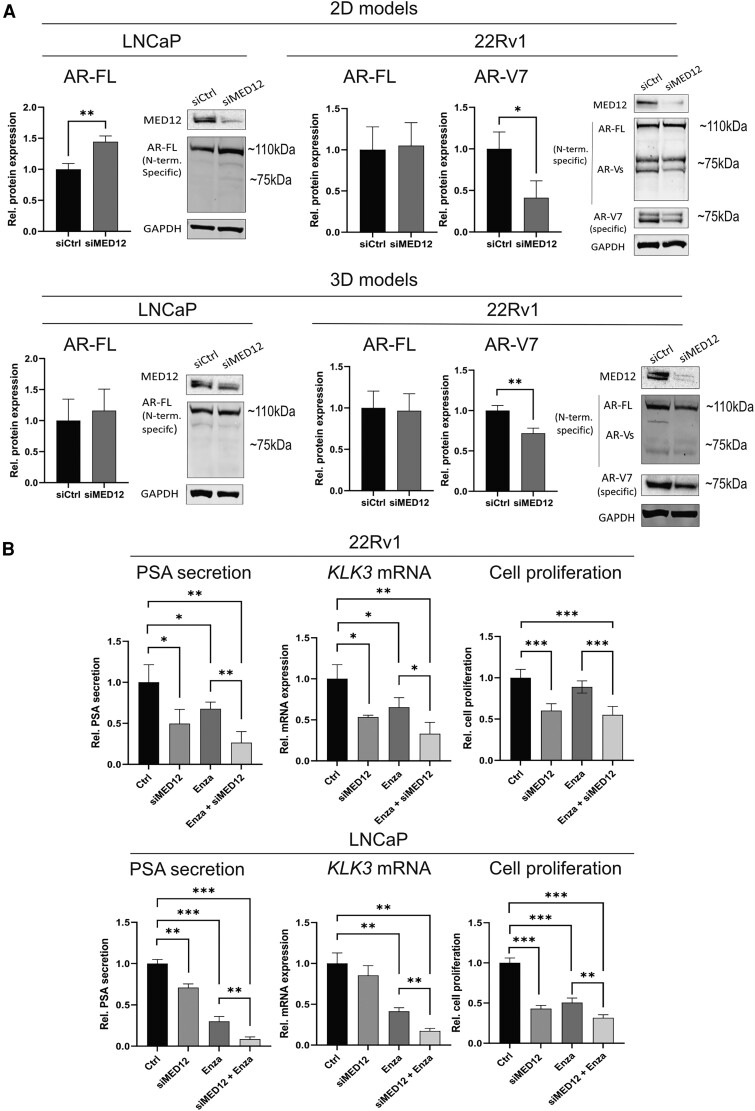
MED12 knockdown inhibits AR-V7 protein expression and increases enzalutamide efficacy. (A) AR-FL and AR-V7 protein expression in LNCaP and 22Rv1 cells upon MED12 knockdown [transfection time in 2D models: 6 days (22Rv1), 3 days (LNCaP); transfection time in 3D models: 7 days]. A representative Western blot is shown for each cell line. (B) Effect of combined siMED12 and 10 µM enzalutamide treatment on prostate-specific antigen secretion medium (chemiluminescent-based assay), *KLK3* mRNA (quantitative reverse transcription polymerase chain reaction), and cell proliferation (fluorescent-based nuclei counting) in 22Rv1 and LNCaP cells. All data are presented as mean ± SD. Statistical significance: *P < .05, **P < .01, ***P < .001.

We investigated whether MED12 knockdown influences AR protein expression and alternative splicing. Full-length AR (AR-FL) protein expression was unaffected in the LNCaP spheroids and showed increased expression in the 2D setting (+44%; *P* = .004) (Fig. 4A). LNCaP cells do not express AR-V7 protein and MED12 knockdown did not induce its expression. In 22Rv1 cells, MED12 inhibition significantly downregulated AR-V7 mRNA and protein expression (−59%, *P* = .02) without affecting AR full-length (AR-FL) protein expression in both 2D and 3D models [Supplementary Fig. S5 (25), Fig. 4A].

Since AR-V7 promotes enzalutamide resistance (7), we investigated whether siMED12-mediated downregulation of AR-V7 protein influences 22Rv1 cell sensitivity to enzalutamide (2D models). The combination of MED12 knockdown and enzalutamide treatment after 6 days additively decreased PSA protein secretion and *KLK3* mRNA expression compared with enzalutamide treatment alone (Fig. 4B). However, the combination treatment did not enhance the antiproliferative effects of the siMED12 single treatment at this relatively short time point (Fig. 4B). To clarify the inconsistency between AR activity and proliferation, we examined early cell responses [Supplementary Fig. S6 (25)]. MED12 knockdown resulted in a rapid and sustained decrease in *KLK3* and *c-MYC* mRNA expression levels. However, we did not observe consistent effects on *AR-V7* mRNA expression at these early time points. Similarly, enzalutamide treatment did not affect AR activity in 22Rv1 cells during the first 72 hours. This suggests that observing additive effects on cell proliferation might require longer treatment durations than the 6 days achieved with transient siRNA-mediated knockdown.

To further explore the relationship between MED12 and enzalutamide, we performed the same experiments in LNCaP cells, which are sensitive to enzalutamide and do not express the AR-V7 protein. MED12 knockdown single treatment had weaker effects on AR activity in this cell line than 22Rv1. The combination of MED12 knockdown and enzalutamide treatment significantly decreased PSA protein secretion and *KLK3* mRNA gene expression compared to enzalutamide treatment alone [Fig. 4B, Supplementary Figure S7 (25)]. This confirmed that MED12 knockdown and enzalutamide play an additive or synergistic role in AR inhibition, even in the absence of AR-V7 expression. Conversely, *AR-V7* and *c-Myc* mRNA expression levels were not additively altered at 48 and 72 hours of treatment [Supplementary Fig. S7 (25)]. After 6 days, the combination of siMED12 and enzalutamide resulted in a small but significant decrease in cell proliferation compared to enzalutamide alone (Fig. 4B).

### CDK8/19 Inhibition Decreases Proliferation in Prostate Cancer Cell Lines

MED12 is a fundamental structural component of the kinase module of the Mediator complex (27). Its disruption impairs the enzymatic activity of the whole module exerted by CDK8 or its alternative paralog CDK19 (17). Thus, we hypothesized that MED12 knockdown affects prostate cancer cell lines mainly through the subsequent loss of the kinase module activity. This suggests that the direct inhibition of CDK8/19 may affect prostate cancer cells similarly to what was observed with MED12 loss.

**Figure 5.**
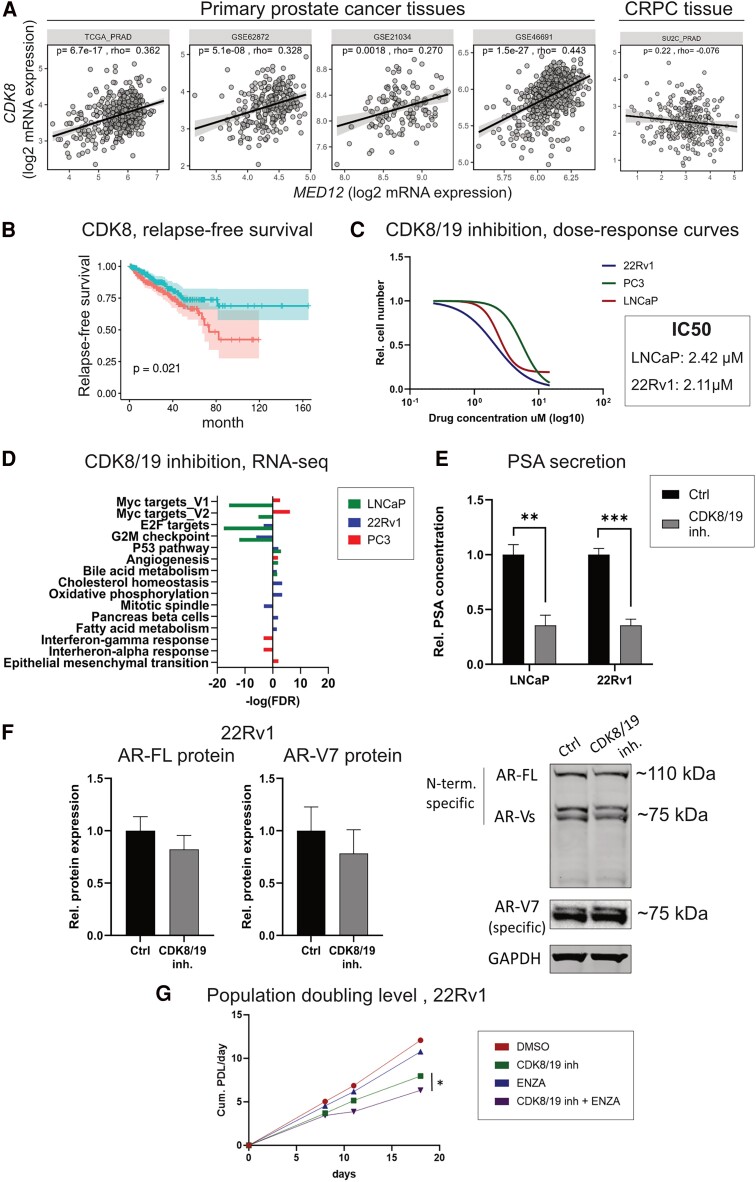
CDK8/19 inhibition and enzalutamide additively decrease cell proliferation in 22Rv1. (A) MED12 and CDK8 gene expression correlation in cohorts of primary (TCGA_PRAD, GSE62872, GSE21034, GSE46691) and castration-resistant prostate cancer tissues (SUC2_PRAD). (B) Relapse-free survival of treatment-naïve prostate cancer patients (TCGA database) with high (media = above) and low (media = below) CDK8 expression. (C) Dose-response curves of SEL-120-34A (CDK8/19 inhibitor) in LNCaP, 22Rv1, and PC3 (fluorescent-based assay, treatment time: 3 days). The IC50 for cell proliferation was reported. (D) MSigDB cancer hallmarks significantly altered in LNCaP, 22Rv1, and PC3 cells upon CDK8/19 inhibition (treatment time: 3 days). Statistical significance: false discovery rate < 0.05. (E) Quantification of prostate-specific antigen secreted by LNCaP and 22Rv1 treated for 3 days with CDK8/19 inhibitor (SEL-120-34A) or Ctrl (dimethylsulfoxide) (chemiluminescent-based assay). (F) Quantification of AR-FL and AR-V7 protein expression in 22Rv1 cells treated for 3 days with CDK8/19 inhibitor (SEL-120-34A) or Ctrl (dimethylsulfoxide). A representative Western blot is shown for each cell line. Data are presented as mean ±SD. (G) Cumulative population doubling level of cells treated with: Ctrl (dimethylsulfoxide); 1.5 µM CDK8/19; 10 µM enzalutamide; 1.5 µM CDK8/19 and 10 µM enzalutamide (number of replicates: 1). All data are presented as mean ±SD. Statistical significance: *P < .05, **P < .01, ***P < .001. Abbreviation: CDK, cyclin-dependent kinase.

*MED12* mRNA expression was positively correlated with *CDK8* and *CDK19* gene expression in multiple primary prostate cancer tissue datasets, as expected by their common presence in the kinase module. However, no significant correlation was detected in the CRPC tissue database [Fig. 5A, Supplementary Fig. S8 (25)]. High CDK8 expression significantly decreased relapse-free survival in treatment-naïve patients with prostate cancer (TCGA database), whereas CDK19 expression did not [Fig. 5B, Supplementary Fig. S9 (25)]. In prostate cancer cell lines, CDK8 protein expression was higher in PC3 cells than in LNCaP and 22Rv1 cells. Consistent with the alternative roles of CDK8 and CDK19 within the Mediator complex, CDK19 was expressed at lower levels in PC3 cells than in the other cell lines. All these expression trends were mirrored by their relative mRNA expression [Supplementary Fig. S10 (25)].

SEL120-34A is a CDK8/19 subunit inhibitor that reduces tumor growth in acute myeloid leukemia (28). A phase 1B clinical trial (NCT04021368) is currently underway to assess its pharmaceutical potential. We treated LNCaP, 22Rv1, and PC3 cells with SEL120-34A to test their sensitivity to biochemical CDK8/19 inhibition. Consistent with observations for MED12, AR-positive cells were slightly more sensitive to CDK8/19 inhibition than the AR-negative PC3 cells (IC50: LNCaP—2.42 µM; 22Rv1—2.11 µM; PC3—5.6 µM) (Fig. 5C). High-throughput 3′ RNA-sequencing and pathway analysis revealed that CDK8/19 inhibition downregulated the c-Myc pathway, E2F targets, and G2 M checkpoint hallmarks in at least 1 cell line (Fig. 5D). Conversely, it upregulated the tumor-suppressor p53 pathway in AR-positive cells (Fig. 5D).

Mirroring the effects of MED12 knockdown, CDK8/19 inhibition decreased PSA secretion in LNCaP (−64%; *P* = .001) and 22Rv1 cells (−64%; *P* = .0001) after 3 days of treatment (Fig. 5E). Notably, AR-V7 protein expression remained unchanged at this early stage (Fig. 5F), further underscoring the need for extended time points to assess potential synergy between CDK8/19 inhibition and AR. Consequently, we cotreated 22Rv1 cells with SEL120-34A and enzalutamide for 18 days. By day 8, the population doubling in the double-treated cells began to decline, and the drug combination demonstrated a significant overall effect over the time course (*P*-value for CDK8/19 inhibition + ENZA vs CDK8/19 inhibition: 0.038) (Fig. 5G). Taken together, these findings suggest that CDK8/19 inhibition modulates the response to enzalutamide in 22Rv1 cells.

## Discussion

Although elements of the Mediator complex have been implicated in multiple oncogenic processes, our knowledge of their specific functions in prostate cancer is limited. Our findings revealed that the downregulation of MED12 inhibits c-Myc signaling and cell proliferation. We showed that MED12 and CDK8/19 inhibition downregulate the AR response and that they both affect prostate cancer cell response to enzalutamide.

These results align with previous studies associating MED12 and CDK8/19 inhibition with reduced cell proliferation (18, 20, 29). Although our study is the first to relate MED12 to c-Myc in prostate cancer, MED12 depletion was already related to c-Myc expression in colon cancer (30). Our RNA-seq analysis revealed that CDK8/19 inhibition differentially modulates c-Myc signaling based on the in vitro model. Previous studies have positively correlated CDK8 and c-Myc expression in cancer (20, 29, 31). While this manuscript was in preparation, Li and colleagues reported that inhibition of the Mediator complex kinase under castration conditions downregulated the Myc pathway in xenograft models of prostate cancer (29). Thus, the results of our study, along with those of Li et al, establish a basis for therapeutic intervention targeting the kinase module of the Mediator complex. c-Myc activation can modulate cell proliferation through various mechanisms, including the regulation of mitochondrial functions (32) and the metabolic changes resulting from AR inhibition (33). For example, c-Myc maintains the glycolytic activity (33) and increases the expression of key fatty acid synthesis genes, such as ATP citrate lyase, acetyl-CoA carboxylase alpha, and fatty acid synthase (34), thus promoting prostate cancer progression (35).

MED12 knockdown and CDK8/19 inhibition downregulated the AR activity in our 3D models. Other researchers have also recognized the value of 3D models for studying the androgenic regulation in vitro (36, 37). MED12 affected the AR target genes *FKBP5* and *TMPRSS2* to a lesser extent than *KLK3*. This differential regulation may reflect the variable AR cofactors that are required for their expression. For example, the cofactors NCOA2 and NCOA7 are important modulators of *KLK3* mRNA expression in VCaP cells (38), whereas *TMPRSS2* expression can occur in an AR-independent manner (39).

Surprisingly, MED12 inhibition increased AR-FL expression in LNCaP cells. This could indicate a compensatory mechanism of the cells or suggest that MED12 might play a dual role depending on the cancer stage. However, it is noteworthy that combination with enzalutamide led to an additive effect. In 22Rv1 cells, MED12 knockdown consistently decreased AR-V7 protein expression, while AR-FL expression remained stable. AR-V7 is upregulated during antiandrogen resistance and modulates the proliferation and motility of prostate cancer cells (40), as well as cell sensitivity to cabazitaxel (40). Recent studies have also revealed that AR-V7 does not merely mimic AR-FL activity but can activate a unique set of target genes (41, 42).

We demonstrated that CDK8/19 inhibition partially resensitized 22Rv1 cells to enzalutamide. This finding aligns with the study of Offermann et al, which showed that CDK8/19 inhibition combined with bicalutamide, an AR inhibitor, additively reduced cell proliferation (22).

To our knowledge, MED12 and CDK8/19 may influence the androgen and enzalutamide responses through both AR-V7-dependent and -independent mechanisms. Some Mediator complex subunits, such as MED1 and MED19, are cofactors of AR and enhance its transcriptional activity in prostate cancer (9, 43). However, MED12 and CDK8/19 belong to the kinase module of the Mediator complex; therefore, their inhibition may disrupt the phosphorylation events that modulate the activity of AR and its variants (AR-Vs). Multiple kinases are already known to phosphorylate AR and AR-Vs, thus modulating their activity and protein stability (44). Considering the involvement of c-Myc in AR activity and enzalutamide resistance, MED12 may also influence these processes in a c-Myc-mediated manner (45). c-Myc overexpression contributes to suppression of AR activity, with low AR activity and high c-Myc being typical signatures of aggressive prostate cancer (46). According to our RNA-seq data, MED12 knockdown inhibits OXPHOS. Since enzalutamide-resistant cells primarily rely on OXPHOS for their growth, MED12-mediated regulation may increase the cell sensitivity to enzalutamide (47).

The MED12 and CDK8/19 subunits not only belong to the kinase module but are also essential for the kinase activity (16, 17, 48). Consistent with this knowledge, we observed that their inhibition partially altered the same cancer-promoting pathways (eg, the Myc pathway, E2F targets, and G2M checkpoints). However, these alterations did not completely overlap. An example is the P53 pathway, which is upregulated only following CDK8/19 inhibition. A potential explanation for this differential outcome may be the Mediator complex-independent functions of MED12. For instance, Huang et al showed that MED12 can translocate to the cytoplasm and regulate the TGF-β receptor (49). This phenomenon might be amplified by the overexpression of MED12 observed in advanced prostate cancer by Adler et al (18).

Our findings regarding the impact of CDK8/19 on prostate cancer suggest promising avenues for therapeutic interventions. SEL120-34A, the CDK8/19 inhibitor used in our study, is currently in a phase 1B clinical trial (NCT04021368) for acute myeloid leukemia. Although in vivo studies are beyond the scope of our research, our results may provide the basis for in vivo assessment of SEL120-34A feasibility for prostate cancer treatment.

Despite providing novel insights into the role MED12 and CDK8/19 in prostate cancer, our study has several limitations. Future research should aim to determine the effects of MED12 and CDK8/19 inhibition on AR signaling and enzalutamide responses over an extended period. Clarifying the roles of MED12 and CDK8/19 in modulating AR and AR-Vs is crucial for understanding their impact on prostate cancer. Addressing these questions requires further study on other AR-positive cell lines.

In conclusion, our study highlights the involvement of MED12 and CDK8/19 in c-Myc, AR activity, and cell response to enzalutamide in prostate cancer. These findings provide a valuable understanding of potential therapeutic targets for future research.

## Data Availability

Datasets generated and/or analyzed in the current study are available in the NCBI GEO database using accession numbers GSE269070, GSE269071, GSE269072, and GSE269 073.
